# Combination of tauroursodeoxycholic acid, co-enzyme Q10 and creatine demonstrates additive neuroprotective effects in *in-vitro* models of Parkinson’s disease

**DOI:** 10.3389/fnins.2024.1492028

**Published:** 2024-12-23

**Authors:** Alexander Shtilbans, Wolfgang E. Reintsch, Valerio E. C. Piscopo, Andrea I. Krahn, Thomas M. Durcan

**Affiliations:** ^1^Hospital for Special Surgery, New York, NY, United States; ^2^Weill Cornell Medicine, New York, NY, United States; ^3^The Neuro’s Early Drug Discovery Unit (EDDU), McGill University, Montreal, QC, Canada

**Keywords:** iPSC cells, combination therapy, Parkinson's disease, *in vitro* model, neuroprotection

## Abstract

This study aimed to evaluate different combinations of three dietary supplements for potential additive or synergistic effects in an *in vitro* Parkinson’s Disease model. The complex and diverse processes leading to neurodegeneration in each patient with a neurodegenerative disorder cannot be effectively addressed by a single medication. Instead, various combinations of potentially neuroprotective agents targeting different disease mechanisms simultaneously may show improved additive or synergistic efficacy in slowing the disease progression and allowing the agents to be utilized at lower doses to minimize side effects. We evaluated four possible combinations of the three selected supplements: tauroursodeoxycholic acid (TUDCA), co-enzyme Q10 (CoQ10), and creatine, chosen for their effects on different targets that had previously shown neuroprotective effects in preclinical models. We evaluated the following combinations: (1) TUDCA+CoQ10, (2) TUDCA+Creatine, (3) CoQ10 + Creatine, and (4) TUDCA+CoQ10 + Creatine. We used induced pluripotent stem cell (iPSC) derived human dopaminergic neurons from a patient with Parkinson’s disease and healthy control, as well as microglial cells, to evaluate for an additive or synergistic effect of these combinations on neurodegeneration and neuroinflammation. We used neurofilament heavy chain, tubulin filament, and proinflammatory cytokines as metrics. We have identified a triple combination of these supplements that showed an additive protective effect across all these endpoints. Indeed, the agents in that combination could address the majority of the known pathways leading to neurodegeneration, such as accumulation of misfolded *α*-synuclein, mitochondrial dysfunction, reactive oxygen species, and neuroinflammation. We demonstrated that the combination of TUDCA, CoQ10, and creatine exerts an additive effect in *in vitro* models of a neurodegenerative disease, surpassing the efficacy of each compound individually. This combination shows strong potential as a candidate for further preclinical confirmatory studies and clinical trials as a neuroprotective treatment for patients with, or at risk for, Parkinson’s disease.

## Introduction

**Figure 1:**
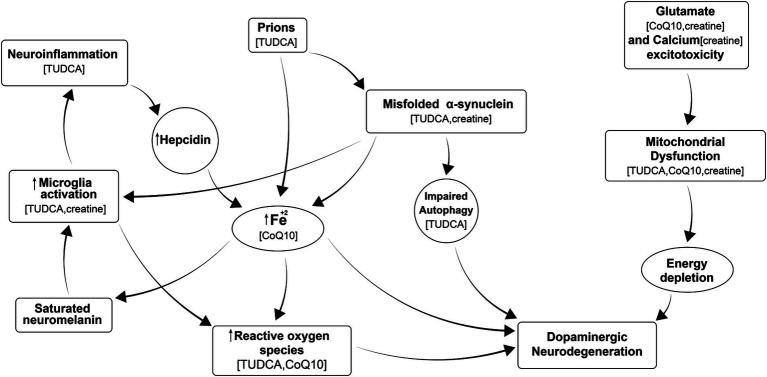
Multiple processes leading to neurodegeneration in PD include: misfolding of proteins that fail to be cleared from the brain, which in turn stimulates neuroimmune responses; calcium excitotoxicity, affecting mitochondria and resulting in energy depletion, leading to neurodegeneration; iron accumulation that leads to activation of microglia and further neuroinflammation, causing oxidative stress, formation of reactive oxygen species (ROS); and mitochondrial dysfunction, and neuroinflammation all of which ultimately contributes to neurodegeneration. Supplements in parenthesis are thought to inhibit the corresponding process.

There is a critical need to develop efficient therapies to combat Parkinson’s disease (PD) and other neurodegenerative diseases (NDs) and also to delay the onset of these diseases in populations at risk. Existing medications only provide improvement for some symptoms of the disease and often require increased doses as the disease progresses, causing side effects. Despite global research efforts, there are still no neuroprotective or disease-modifying drugs for PD, and nothing is available to prevent it. However, we know that processes leading to neurodegeneration include (a) misfolding of proteins that fail to be cleared from the brain, which in turn stimulates neuroimmune responses; (b) calcium excitotoxicity, affecting mitochondria and resulting in energy depletion, leading to neurodegeneration; (c) iron accumulation that leads to activation of microglia and further neuroinflammation, causing oxidative stress, and formation of reactive oxygen species (ROS); (d) mitochondrial dysfunction; and (e) neuroinflammation, as shown in Figure 1 (Cuervo et al., 2004; Mosharov et al., 2009; Ryan et al., 2013; Zucca et al., 2017; Hurley et al., 2013; Rocha et al., 2018). All of these processes occur in each patient at different times and at different rates. Therefore, it is difficult to test a single medication to improve just one of these processes in a clinical trial using a heterogeneous PD patient population, as we do not know that all participants have the same degree of dysfunction in a particular targeted process at a given time. One solution is to use personalized medicine, in which we identify patients with predominantly dysfunctional processes and use a particular medication to treat them. Alternatively, we propose that a “cocktail” of different medications or supplements should be used containing individual agents to address each of the abovementioned pathways, leading to neurodegeneration in the general PD patient population. This proposed approach to using a combination of different medications appears more realistic, as it could be applied to all patients suffering from that disorder.

The vast majority of basic science and clinical research in the field of ND has been devoted to studying individual drugs that can improve single molecular mechanisms leading to neurodegeneration (Vijiaratnam et al., 2021). Thus far, examples from medicine, such as in HIV and certain cancers, have shown that some conditions can only be effectively controlled with a “cocktail” of different medications.

Research studies on certain supplements reported significant positive outcomes in PD models at least preclinically. Therefore, we selected three existing nutritional supplements for this study to be tested in different combinations: tauroursodeoxycholic acid (TUDCA), coenzyme Q10 (CoQ10), and creatine. They have been previously evaluated individually *in vitro* and *in vivo* for some of the NDs and showed promising efficacy in preclinical models of PD, but either failed to demonstrate clinical evidence of disease modification/neuroprotection in clinical trials or have not been studied in patients with PD. They were chosen for their different mechanisms of action targeting various processes that lead to neurodegeneration, as in Figure 1. All of these supplements cross blood–brain barrier and engage a target: CoQ10 (Matthews et al., 1998), creatine (Ohtsuki et al., 2002) and TUDCA (Parry et al., 2010).

### TUDCA

Tauroursodeoxycholic acid (TUDCA), an endogenous bile acid, is a potent neuroprotective agent. TUDCA is a taurine conjugate of ursodeoxycholic acid (UDCA). Duan et al. showed that the application of TUDCA facilitates the survival of DA neurons in *in vitro* and *in vivo* conditions (Duan et al., 2002). The TUDCA-treated group demonstrated an increase in the number of tyrosine hydroxylase-positive neurons, used as a marker for dopamine, norepinephrine, and epinephrine-containing neurons (Weihe et al., 2006), and a reduction in the number of apoptotic cells. In the 1-methyl-4-phenyl-1, 2, 3, 6-tetrahydropyridine (MPTP) mouse model, pretreatment with TUDCA (50 mg/kg for 3 days) significantly reduced neurodegeneration of the nigral dopaminergic neurons caused by MPTP. Additionally, TUDCA treatment reduced dopaminergic fiber loss and ameliorated motor performance, including spontaneous activity, the ability to initiate movement, and tremors, all of which are characteristic symptoms of PD (Castro-Caldas et al., 2012). In the same study, the TUDCA treatment also prevented the production of MPTP-dependent ROS (Castro-Caldas et al., 2012). In the chronic mouse model of PD, pretreatment with TUDCA prevented protein oxidation and autophagy, in addition to inhibiting *α*-SYN aggregation (Cuevas et al., 2022). TUDCA-dependent mitoprotective effects have also been observed in primary mouse cortical neurons and the neuroblastoma cell line SH-SY5Y (Rosa et al., 2017). These findings highlight TUDCA’s potential in attenuating mitochondrial dysfunction, reducing ROS production, and inhibiting multiple proteins involved in apoptosis. Additionally, TUDCA has anti-inflammatory properties in the brain due to its binding to G protein-coupled bile acid receptor 1/Takeda G protein-coupled receptor 5 (GPBAR1/TGR5) in microglia. This results in the secretion of intracellular cAMP levels in microglia and the reduction of proinflammatory biomarkers (Yanguas-Casás et al., 2017).

### CoQ10

Coenzyme Q10 (CoQ10), also known as ubiquinone, is produced in the body and helps various cellular processes. In PD, a study with different dosages of CoQ10 showed that higher doses might slow disease progression, indicated by a lesser increase in scores on the Unified Parkinson Disease Rating Scale (UPDRS) (Shults et al., 2002). Various formulations of CoQ10 have been tested for their neuroprotective effects against dopaminergic neuron loss in PD models, revealing that CoQ10, especially when reduced (ubiquinol), can significantly increase plasma concentrations and provide neuroprotection (Cleren et al., 2008). Interestingly, the prophylactic application of water-soluble CoQ10 formulations in rat models showed promising results in preventing dopaminergic neurodegeneration and behavioral impairment (Somayajulu-Niţu et al., 2009).

Another study showed the potential of prophylactic and therapeutic CoQ10 treatment in a PQ-induced PD mouse model. They reported that CoQ10 effectively reduced protein carbonyl content in the brain and improved behavioral outcomes (Attia and Maklad, 2018). A preclinical study determined a neuroprotective role of coenzyme Q10 in iron-induced apoptosis in cultured human dopaminergic (SK-N-SH) neurons, in metallothionein gene-manipulated mice, and in *α*-synuclein knockout mice with a primary objective to assess a possible therapeutic potential for CoQ10 in PD (Kooncumchoo et al., 2006). Another study demonstrated that continuous, intrastriatal administration of CoQ10 could prevent dopaminergic neuron degeneration more effectively than oral administration, suggesting a new strategy for neurodegeneration prevention in PD (Park et al., 2020). However, high doses of CoQ10 failed to show any disease-modifying effects in a randomized, double-blind, placebo-controlled clinical trial in PD (Beal et al., 2014).

Interestingly, studies have shown that CoQ10, combined with other supplements such as creatine, can provide additive neuroprotective effects in various neurodegenerative disease models. This combination has been effective in reducing oxidative stress, DNA damage, and neurodegeneration in animal models of PD and Huntington’s disease (Yang et al., 2009).

### Creatine

Creatine (CR), commonly recognized as an ergogenic aid for sports and exercise, has shown promising neuroprotective properties against different neurodegenerative conditions in preclinical models. In PD, a clinical trial examined the combined effect of creatine and coenzyme Q10 (CoQ10) on mild cognitive impairment in PD (PD-MCI). The patients were randomly treated with creatine monohydrate (5 g twice a day) and CoQ10 (100 mg three times a day) or a placebo. The study found that after 12 and 18 months, the combination therapy group showed significant improvements in cognitive scores and reduced plasma phospholipid levels compared to the control group, suggesting a neuroprotective function of this therapy in PD-MCI patients (Li et al., 2015). Another study focusing on the therapeutic effects of resistance training with and without creatine supplementation in patients with mild to moderate PD revealed that creatine supplementation enhanced the benefits of resistance training. This was evident in increased chest press, and biceps curl strength and improved chair rise performance, indicating improved muscular fitness and functionality in PD patients (Hass et al., 2007). However, high doses of creatine failed to show any disease-modifying effects in a long, randomized, double-blind placebo-controlled clinical trial in PD (Kieburtz et al., 2015). Moreover, in a mouse model of PD, the combined administration of cyclooxygenase 2 (COX-2) inhibitor rofecoxib and creatine showed significant neuroprotective properties. They effectively protected against striatal dopamine depletions and loss of substantia nigra tyrosine hydroxylase immunoreactive neurons, suggesting the potential of creatine as part of a combined therapeutic strategy for PD (Klivenyi et al., 2003). Furthermore, both creatine and exercise had anti-inflammatory and antioxidant effects in MPTP mice, as evidenced by a decrease in microglia activation (Leem et al., 2024).

To test all combinations of the aforementioned three supplements for possible additive/synergistic effects, we used *in vitro* PD and neuroinflammation models as examples. We studied the effects of these combinations on iPSC cells derived from dopaminergic neurons of a patient with idiopathic PD and healthy control. Then, we used human microglial cells to test the same combinations for potential antineuroinflammatory effects.

The collective published findings on TUDCA, CoQ10, and creatine highlight their possible additive therapeutic potential in PD. To prove this, we chose an *in vitro* model of Parkinson’s disease as an example to explore the potential advantage of combining these supplements to achieve better efficacy.

## Materials and methods

The use of human iPSCs and iPSC-derived cells in this research was approved by the McGill University Research Ethics Board (IRB Study Number A03-M19-22A). The cell lines used were from McGill’s C-BIG Repository—a collection of biospecimen samples, clinical information, imaging, and genetic data from individuals with neurological diseases and healthy control subjects. No special recruitment was conducted specifically for this study, but the tissues in the repository have been collected since 1 March 2016, and it is still ongoing. All individuals who donated material for iPSC cell lines were over 21 and provided written informed consent. The samples from the repository for the current research study were assessed between 25 July 2023 and 22 September 2023.

### Subjects

The Parkinson’s Disease line QPN989 was generated from the peripheral blood mononuclear cells (PBMC) of a Caucasian female patient with sporadic idiopathic Parkinson’s disease (PD). The patient from whom PBMCs were collected was 42 years old. There was no known family history of PD. Sequencing of common PD-associated genes (SNCA, PARK2, PARK6, GBA1, LRRK2, TMEM175, MAPT) revealed no mutations/variants, and no genetic cause was attributed to the patient. No brain imaging data were available.

The healthy control cell line QPN929 was generated from the PBMCs of a healthy 41-year-old Caucasian female with no family history of PD. No mutations were observed in the healthy control subject either.

### Dopaminergic cell lines

iPSCs were reprogrammed from PBMCs with episomal plasmids. Once iPSC clones were obtained, they underwent a complete quality control workflow before being made available for use. Quality control included short tandem repeat (STR) profiling, genome stability testing, karyotyping, pluripotency checks, mycoplasma tests, and trilineage tests. The QC process was described in an earlier study (Chen et al., 2021). Dopaminergic neural progenitor cells (DA NPCs) generated from the patient and the healthy control were seeded in an automated manner into 96-well plates (15,000 cells seeded per well) in the DA differentiation medium and allowed to attach for 24 h. The seeding of cells was automated using an EL-406 Washer Dispenser (BioTek)^1^ for plate coating, cell seeding, fixing, and staining, as described in Krahn et al. (2020). Once seeded, DNs were matured for 2 weeks in maintenance media as previously described (Chen et al., 2019; Chen et al., 2022). Six replica plates were seeded per cell line. We used 12 wells per sample tested. Cells were only seeded into the inner 60 wells of each 96-well plate to avoid potential edge effects that could skew the results. After 2 weeks of differentiation, half of the medium was exchanged with fresh medium, and compounds/combos were added (TUDCA, CoQ10, creatine, and four combos: Creatine+CoQ10, TUDCA+CoQ10, TUDCA+Creatine, and TUDCA+Creatine+CoQ10, also untreated PD cells as control), using a contact-free automated droplet dispenser (I.DOT, Dispendix)^2^. First, we evaluated the solubility of the compounds in testing media and the absence of toxic effects of the drugs and the media on dopaminergic cells. The drug doses used were based on published literature and our prior testing experiments, and are as follows: Creatine: 10 μM (Cunha et al., 2013); CoQ10: 10 μM (Cooper et al., 2012), and TUDCA: 50 μM (Duan et al., 2002). All compounds were added simultaneously, and the treatment time was kept constant. All dosages were provided at one concentration. After 7 days, a half medium exchange and compound/combo addition were repeated. Another 4 days later, this exchange/addition was repeated (a total of 3 cycles of treatments). After 4 more days, dopaminergic neurons were fixed, stained, imaged, and analyzed.

### Immunofluorescence staining and imaging

The cells were fixed in 2% paraformaldehyde (PFA) in phosphate-buffered saline (PBS) at room temperature (RT) for 10 min, permeabilized with 0.1% Triton X-10S ^3^ for 15 min at RT, and then blocked in 5% bovine serum albumin (BSA)/PBS for 1 h at RT. The cells were incubated with primary antibodies in a blocking buffer overnight at 4°C on a shaker. The primary antibodies were neurofilament heavy chain (N4142, Sigma)^4^, betaIII-tubulin (MAB5564, Millipore)^5^, and MAP2 (CPCA-MAP2, EnCor Biotech)^6^. Secondary antibodies and Hoechst33342 (nuclear counterstain, H3570, Thermo Fisher Scientific)^7^ were applied for 2 h at RT. Images were acquired on an Opera Phenix High Content Imager (Revvity, Waltham, MA, USA), with an X20 NA1.0^8^ water objective in confocal mode, collecting a stack of four images per channel with 4 μm step size.

### Image and data statistical analysis

The images were analyzed using Harmony image analysis software (Revvity)^9^. Before analysis, illumination in all images was corrected, and image stacks were combined using maximum projection. Hoechst33342 stained nuclei were identified as objects to obtain the cell count per well. NFH and beta-tubulin were evaluated in their respective channels by thresholding the image and filtering for cell bodies and filamentous structures (neurites). The identified objects were combined per image for each channel to calculate the total area occupied by neuronal cells per image. The mean per well was calculated as the final output from the per-image data. Further data processing was performed in Excel (Microsoft), combining the replica plates to calculate the mean and standard deviation for each treatment. Each treatment result was normalized against the mean of the vehicle-only control (dimethyl sulfoxide [DMSO]). Statistical significance was tested with a one-way analysis of variance (ANOVA), followed by Bonferroni’s multiple comparison test.

#### Human microglia cell line

We evaluated the antineuroinflammatory effects of CoQ10, Creatine and TUDCA by measuring their effect on proinflammatory cytokines secretion following lipopolysaccharide (LPS) stimulation of human brain microglia cells. The selected doses were chosen based on the published literature and previous experience: CoQ10 = 10 μM; creatine = 10 μM; and TUDCA = 200 μM (Romero-Ramírez et al., 2022; Yanguas-Casás et al., 2017; Kim et al., 2018). AIW002-2-induced pluripotent stem cells (iPSCs) were obtained from a healthy donor as previously described (Chen et al., 2021) and were differentiated into microglia following the described protocol (Dorion et al., 2024). Briefly, iPSCs were plated onto Matrigel-coated 6-well plates on day 1, aiming for a density of 1–5 small colonies. Differentiation into iPSC-derived hematopoietic progenitor cells (iHPCs) was carried out using the STEMdiff™ Hematopoietic kit (STEMCELL Technologies)^10^. iHPCs were collected on days 12 and 14 and plated onto a Matrigel-coated vessel at a density of 5–10 × 10^5^ cells/ml in microglia differentiation medium 2.9 for 28 days. We followed the manufacturer’s protocol for activation of the microglial cells with LPS to subsequently measure the effect of the study drugs on proinflammatory cytokines interleukin 6 (IL-6) and tumor necrosis factor-*α* (TNF-α). All cell culture procedures were performed in aseptic conditions, under a laminar flow hood. Microglia were thawed and cultured following the provider’s instructions. The microglia were plated at 30,000 cells per well of a Corning Primaria 96-well plate (reference number 353872) in 100 μL of growth medium. The cells were incubated at 37°C/5% CO_2_ in a humidified cell culture incubator; 3 days after cell plating, the test or reference substances (25 μL of solutions at 5×) were applied 1 h before LPS stimulation; and 1 h later, 100 ng/mL LPS was added to the cells and incubated for 16 h at 37°C/5% CO_2_ in a humidified cell culture incubator. After LPS incubation, the supernatant of each well was collected and centrifugated at 1000 *g* for 10 min to remove the cells and debris. Fifty microliters of supernatant from untreated and LPS-treated wells were used to determine cytokine secretion (IL-6 and TNF-α) usng the Cytometric Bead Array Human Inflammatory Cytokines Kit (BD Falcon, catalog number 551811) according to the provider’s instructions. Readings were filled on an Attune™ Nxt Flow Cytometer^11^ and analyzed/visualized using FlowJo™ software^12^. Samples were diluted prior to the assay to fit within the standard range if needed. Six wells were used per each condition. Cytokine levels in the supernatant were expressed in pg./ml.

#### Statistical analysis

The results were presented as means with the standard deviation (SD). One-way ANOVA, followed by Dunnett’s multiple comparisons test, was used to analyze the data. All statistical calculations were performed using GraphPad Prism (version 10.2.3)^13^.

## Results

### Effect of experimental conditions on idiopathic PD and healthy control cell lines

While neurofilament light chain measurements are widely used as an extracellular marker for neurodegeneration, measured as protein released from degenerating neurons and accumulating in the cerebrospinal fluid, in this *in vitro* study, we measured neurofilament heavy chain (NFH) as a structural component of the intracellular neuronal cytoskeleton to mark *the healthy neurites*. NFH, *β*-tubulin, and MAP2 are all three markers of neuronal cell bodies and neurites. The goal was to measure the density of the neurite network in culture (measured as the area of an image occupied by neurites). All three markers indicate a change in density with certain treatments.

**Figure 2:**
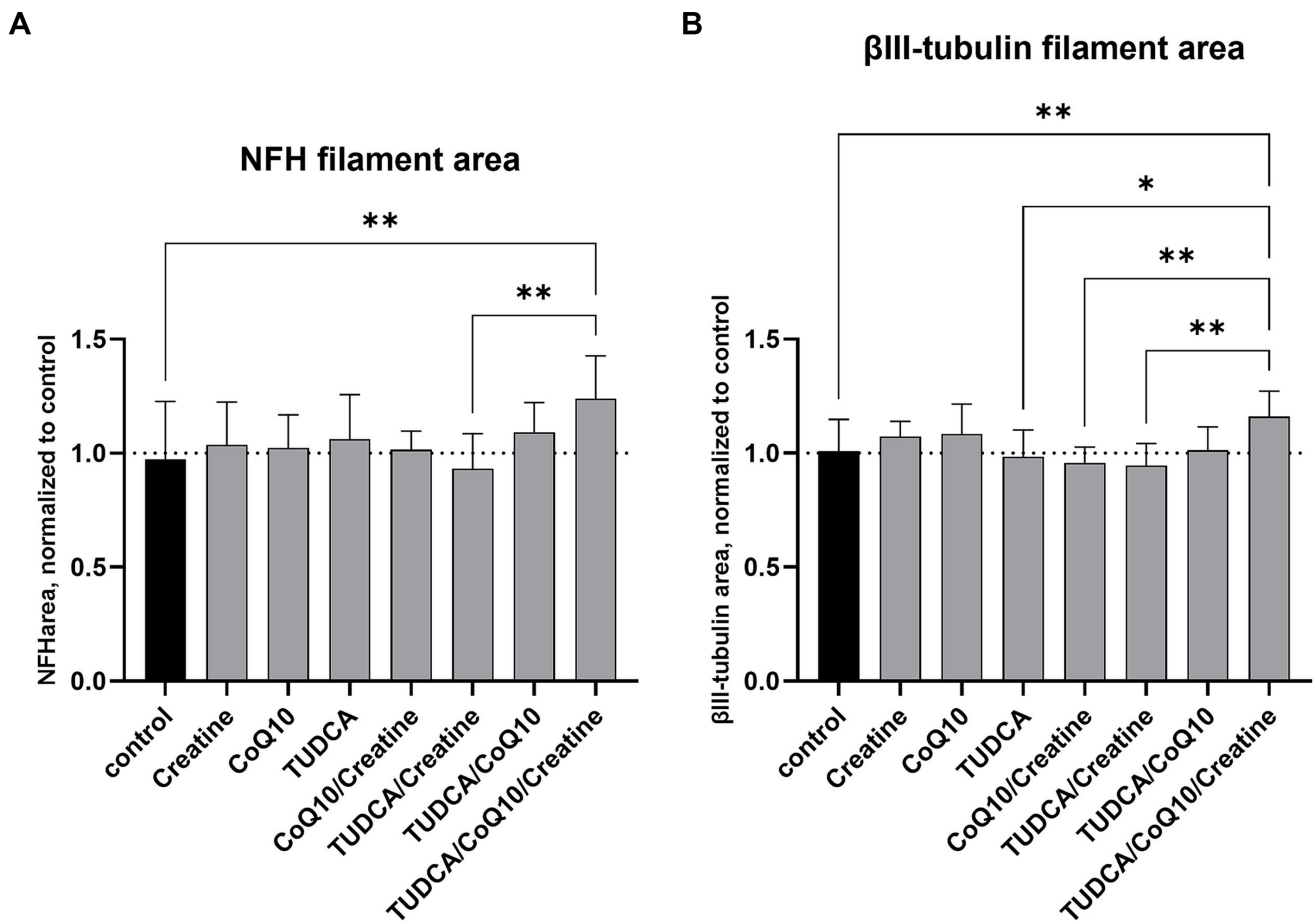
Effects of compound treatment on neural network density of idiopathic PD cell line. **(A)** Neurons are stained for neurofilament heavy chain (NFH) to visualize the neurite network. **(B)** Neurons are stained for βIII-tubulin to visualize the neurite network. **(A,B)** Bars represent the mean surface area of the stained neurite network per well, normalized against the mean area of the control (dimethyl sulfoxide [DMSO] only) wells. Values above 1 after compound treatment indicate an increase in neurite density compared to control. Error bars represent standard deviation. *p < 0.05; **p < 0.001.

The Parkinson’s dopaminergic cell line treated with the combination of TUDCA/creatine/CoQ10 showed a 24% increase in NFH filament area compared to the untreated cells (*p* < 0.001) (Figure 2A). The tubulin filament area was also increased by 16% (*p* < 0.001) compared to the untreated cells (Figure 2B). None of the three individual compounds alone or in other combinations showed an increase in these measures. We did not see any negative or positive changes in the cell number from the treatment with these supplements individually or in any combinations (data not shown).

**Figure 3:**
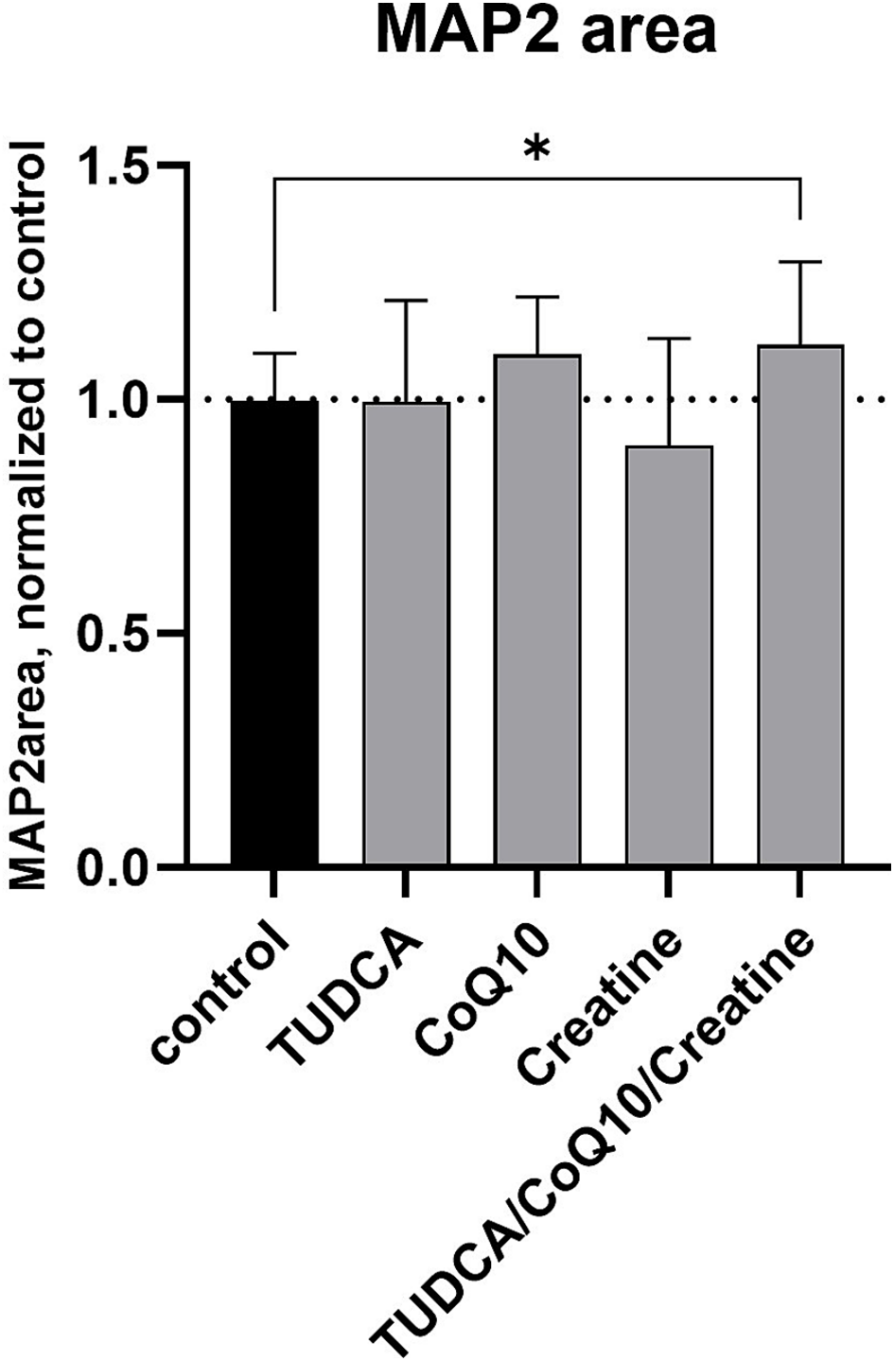
Effects of compound treatment on neural network density of healthy control cell line. Neurons are stained for the neuronal marker MAP2 to visualize the neurite network. Bars represent the mean surface area of the stained neurite network per well, normalized against the mean area of the control (dimethyl sulfoxide [DMSO] only) wells. Error bars represent standard deviation. *p < 0.05.

The healthy control cell line treated with the combination of TUDCA/creatine/CoQ10 showed no changes in the NFH filament area compared to the untreated cells. However, the MAP2 area was increased by 12% (*p* < 0.05) compared to the untreated cells (Figure 3). None of the three individual compounds alone produced a statistically significant increase in these measures. The tubulin filament was not tested. We did not see any negative or positive changes in the cell numbers from the treatment with these supplements individually or in any combinations (data not shown).

### Effect of experimental conditions on human microglia cell line

**Figure 4:**
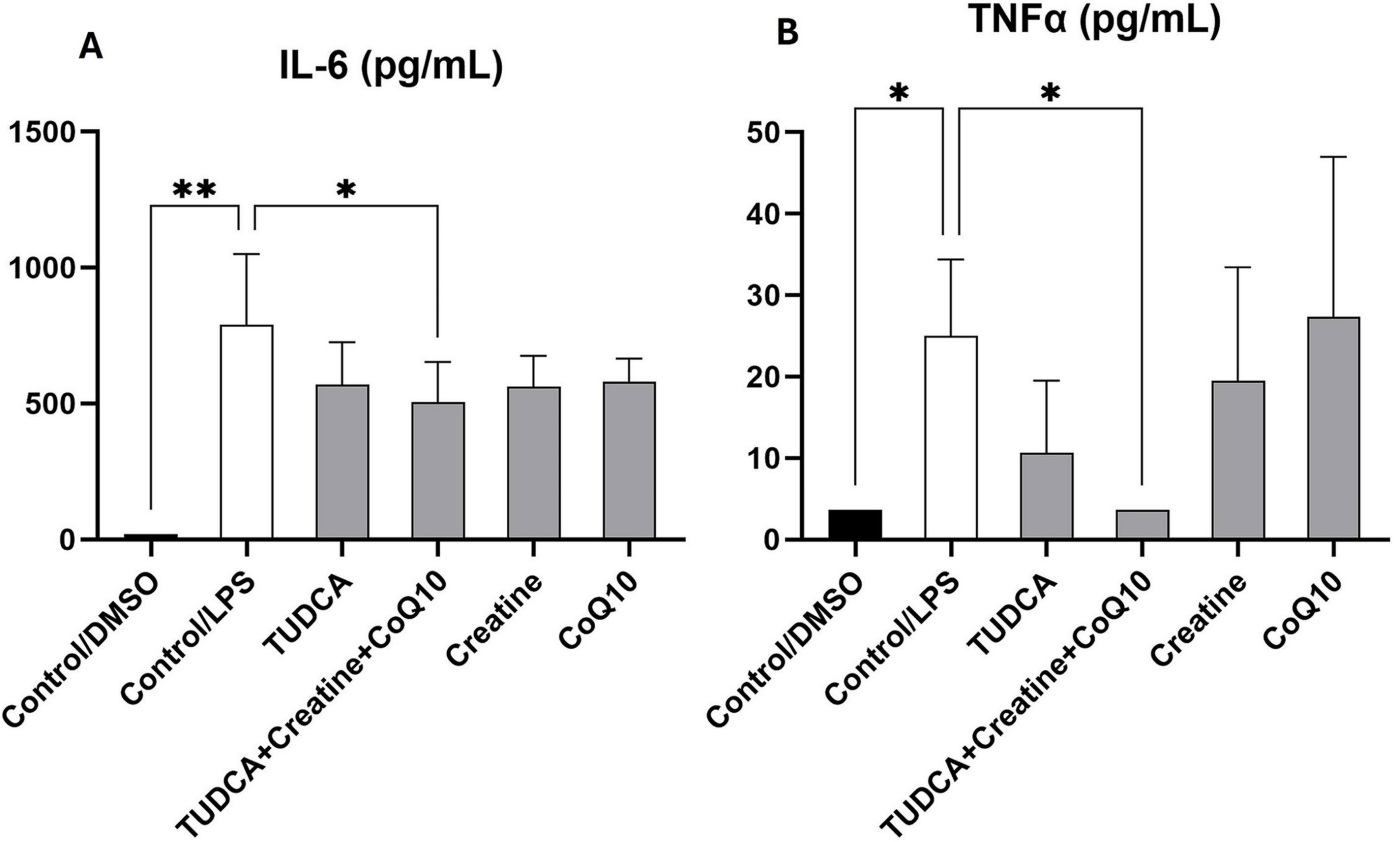
Effect of the experimental conditions on the secretion of proinflammatory cytokines in iPSC-derived microglial cultures. Following treatment, the levels of proinflammatory cytokines interleukin 6 (IL-6; panel **A**) and tumor necrosis factor-*α* (TNF-α; panel **B**) in the supernatants of induced pluripotent stem cell (iPSC)-derived microglia-like cells (iMGLs) was analyzed. The measures are expressed in pg./ml. *p < 0.05; **p < 0.001.

The combination of TUDCA, creatine, and CoQ10 significantly reduced IL-6 levels, demonstrating statistical significance. In contrast, when tested separately, none of the supplements showed a statistically significant reduction of IL-6, although a trend toward reduction was seen (Figure 4A). While TUDCA alone significantly reduced the proinflammatory cytokine TNF-*α*, the triple combination of TUDCA, creatine, and CoQ10 completely abolished TNF-α levels. Neither creatine nor CoQ10 alone resulted in a statistically significant reduction in TNF-α, suggesting a synergistic effect (Figure 4B) of the combination.

## Discussion

We have identified a promising combination of the three aforementioned dietary supplements TUDCA, CoQ10, and creatine, which showed an additive effect on several biomarkers *in vitro* for further preclinical and clinical validation. Indeed, the three tested agents together are thought to affect the majority of the known pathways leading to neurodegeneration in PD, such as aggregation of misfolded *α*-synuclein, neuroinflammation, mitochondrial dysfunction, and ROS formation (Figure 1). This triple combination improved the NFH and tubulin levels in dopaminergic neurons from PD suggesting, if not neuroprotection, but at least enhancement of wellbeing of these cells.

The additive effect of the triple combination observed in this experiment could be explained by: different intracellular targets the individual supplements affect as shown in Figure 1; and/or cumulative additive effects of the supplements on the same targets they share such as mitochondrial function which was not directly evaluated here, unfortunately.

Interestingly, the triple combination resulted in increased MAP2 significantly decreased levels in healthy control dopamine neurons. MAP2 is involved in interactions with cytoskeletal filaments, process formation, synaptic plasticity, and regulation of protein folding and transport. Neuron loss, regardless of cause, is accompanied by a corresponding loss of MAP2 (DeGiosio et al., 2022). Therefore, these supplements, as a combination, should be further evaluated for possible effects on other age-related neurological conditions.

The triple combination of CoQ10, TUDCA, and creatine significantly decreased levels of proinflammatory cytokines, especially TNF-*α*, suggesting a potential reduction of neuroinflammation (Figure 4). Interestingly, this significant reduction in TNF-α was observed only with the triple combination therapy. It was previously reported that a combination of CoQ10 and creatine improved mitochondrial function and cellular bioenergetics and their properties as antioxidants have a role in modulating inflammation. There is a cumulative effect of CoQ10 and creatine that boosts the mitochondria’s ability to prevent inflammation (Yang et al., 2009). Adding TUDCA to the combination most likely further strengthens the anti-inflammatory response as it binds to GPBAR1/TGR5 in the microglia. This results in the secretion of intracellular cAMP levels in the microglia and the reduction of proinflammatory biomarkers (Yanguas-Casás et al., 2017).

TNF-*α* is a central regulator of inflammation in the human body. It is also involved in the pathogenesis of various peripheral autoimmune and inflammatory diseases, including rheumatoid arthritis and inflammatory bowel diseases such as Crohn’s disease and ulcerative colitis. Therefore, the reported triple combination of TUDCA, creatine, and CoQ10 should be further evaluated for these autoimmune conditions that are known to improve from TNF-α inhibition.

This study has several limitations. We only evaluated a cell line from one female patient with Parkinson’s disease, and further confirmation of the findings performed on other cell lines from sporadic and hereditary PD patients is needed. While we are not aware of any gender-specific alterations in the pathways leading to neurodegeneration, it is well known that PD affects more men than women (3:2 ratio) until menopause, after which the ratio becomes 1:1. In certain genetic variants, there could be a predominant pathway involved (neuroinflammation in LRRK2 mutation carriers, or α-synuclein overaccumulation in glucocerebrosidase [GBA] mutation carriers); however, in this study, we used cells from an individual with idiopathic PD, having ruled out all known generic variants. Nonetheless, it would be prudent to reconfirm our findings in male cases in the future to ensure rigor and reproducibility. The microglia experiment was performed on wild-type human microglia activated with LPS and not from PD patients. The model is appropriate as the results could be extrapolated to all NDs, but it would be interesting to confirm the anti-inflammatory effect of the triple combination of TUDCA, creatine, and CoQ10 directly on microglial cells from patients with PD and other NDs. We did not study the effects of other combinations of the three supplements on the microglia, as the first experiment on dopaminergic neurons showed positive effects only from the triple combination. Furthermore, the only improved endpoints from that experiment were NFH and tubulin. We did not see changes in cell count. Evaluation of other biomarkers of cellular well-being, such as mitochondrial function, is also needed. Finally, we focused solely on neurons and microglia as biomarkers and did not evaluate the potential effects of the supplements on astrocytes, which are also involved in neurodegeneration.

Overall, we described a novel *in vitro* screening approach and conceptual design of drug combinations based on their different mechanisms of action, which together could lead to the development of a product capable of providing clinically significant neuroprotection in a major ND. If successful, integrating these three compounds into therapeutic strategies could offer hope for improved treatments and outcomes for patients suffering from PD. As a next step, midbrain organoid models could be applied towards assaying and validating the effects from 2D neurons, in support of advancing these therapeutic strategies into preclinical animal models. Beyond these models, human clinical studies would be certainly needed to confirm the above findings.

## Data Availability

The raw data supporting the conclusions of this article will be made available by the authors, without undue reservation.

## Footnotes

^1^ EL406, sold by Agilent, BioTek EL406 Washer Dispenser. https://www.agilent.com/en/product/cell-analysis/microplate-automation-detection/microplate-washers-dispensers/biotek-el406-washer-dispenser-1623255

^2^I.Dot (Dispendix):https://dispendix.com/idot-non-contact-liquid-handler?cq_src=google_ads&cq_cmp=21140023356&cq_con=&cq_term=&cq_med=&cq_plac=&cq_net=x&cq_plt=gp&utm_term=&utm_campaign=PMAX+Branded&utm_source=adwords&utm_medium=ppc&hsa_acc=4724759833&hsa_cam=21140023356&hsa_grp=&hsa_ad=&hsa_src=x&hsa_tgt=&hsa_kw=&hsa_mt=&hsa_net=adwords&hsa_ver=3&gad_source=1&gclid=CjwKCAiAjKu6BhAMEiwAx4UsAr04C9C765QunKB0J0IZpekYl3zuJk2QVnvT_LVsANOdOerJiy3QXBoCRrMQAvD_BwE

^3^Triton X-100, Sigma Aldrich, Cat# X100. 1X PBS, Wisent, Cat#311-010 CL https://www.wisentbioproducts.com/product/pbs-1x-311-010-cl/ and https://www.sigmaaldrich.com/CA/en/product/sial/x100?srsltid=AfmBOoo8k83VNjgptikaP3kl-HD_IdN5IjPDEcVcT09WepIvKw7bIGY4

^4^Anti-Neurofilament 200 antibody produced in rabbit, Sigma-Aldrich, N4142 https://www.sigmaaldrich.com/CA/en/product/sigma/n4142?srsltid=AfmBOor-RV1Ow5SSeRYGBjZsAYTroVqVYmU5xkAelNvqEteSPxA41vMf.

^5^Anti-Tubulin β III Antibody, EMDMillipore, Cat#MAB5564. https://www.emdmillipore.com/CA/en/product/Anti-Tubulin-III-Antibody,MM_NF-MAB5564?ReferrerURL=https%3A%2F%2Fwww.google.com%2F.

^6^Chicken Polyclonal Antibody to MAP2A/B. Cat# CPCA-MAP2, Encor Biotechnology Inc, https://encorbio.com/product/cpca-map2ab/.

^7^Hoechst 33342, Nuclear Dye, Cat #H3570, ThermoFisher Scientific https://www.thermofisher.com/order/catalog/product/H3570

^8^X20, NA1.0: 20 x objective, numerical objective 1.0, part of Image Xpress purchase.

^9^Harmony High-Content Imaging and Analysis Software https://www.revvity.com/ca-en/product/harmony-5-2-office-revvity-hh17000019.

^10^STEMdiff™ Hematopoietic Kit, Stem Cell technologies, Cat# 05310, https://www.stemcell.com/products/stemdiff-hematopoietic-kit.html.

^11^FlowJo™ v10.10 software, FloJo https://www.flowjo.com/solutions/flowjo.

^12^Prism (version 10.2.3) software, Graphpad, https://www.graphpad.com/updates/prism-10-2-3-release-notes

^13^Attune NxT Flow Cytometer, Thermo FIsher Scientific, https://www.thermofisher.com/ca/en/home/life-science/cell-analysis/flow-cytometry/flow-cytometers/attune-nxt-flow-cytometer/models/nxt.html.
